# Conjunctival Pyogenic Granuloma Management in Bhutan: A Case Report and Literature Review

**DOI:** 10.1155/crop/5561728

**Published:** 2025-12-28

**Authors:** Chencho Gem, Jigme Jamtsho

**Affiliations:** ^1^ Department of Ophthalmology, Central Regional Referral Hospital, Gelephu, Sarpang, Bhutan; ^2^ Department of Pathology, Central Regional Referral Hospital, Gelephu, Sarpang, Bhutan

**Keywords:** benign tumors, capillary hemangioma, conjunctival granuloma, pterygium surgery

## Abstract

**Background:**

Conjunctival pyogenic granuloma is a benign vascular tumor that typically occurs following an eye injury, trauma, after an eye surgery, or during pregnancy due to hormonal changes. The granulomas are commonly seen at the traumatic or the surgical wound site, where improper wound healing leads to the formation of the granuloma. However, in some cases, the cause of the granuloma remains unknown.

**Case Presentation:**

A 47‐year‐old female presented to the Ophthalmology Outpatient Department at the Central Regional Referral Hospital in Gelephu, Bhutan, with a progressively enlarging, reddish mass in her left eye, 2 months after pterygium excision surgery. Ocular examination revealed a pedunculated, vascular lesion on the conjunctiva consistent with a postsurgical conjunctival pyogenic granuloma. The lesion was managed successfully with complete resolution and no recurrence on follow‐up.

**Conclusion:**

This case underscores the need for clinicians to recognize conjunctival pyogenic granuloma as a potential postoperative complication following pterygium excision. Early diagnosis and appropriate management can prevent recurrence and other related complications. To our knowledge, this represents one of the first such cases reported from our hospital in the past 5 years.

## 1. Introduction

Conjunctival pyogenic granuloma, also known as a lobular capillary hemangioma or a reactive hemangioma, is an inflamed vascular tumor that consists of capillary proliferations [1, 2] and occurs from the mucous membranes/conjunctiva and is benign in nature [3]. They develop after an eye injury or eye‐related surgeries like chalazion removal, strabismus surgery, pterygium excision, or enucleation [3–5]. In some cases, the cause remains unknown, without any significant history. These lesions usually appear a few weeks after an insult to the intact conjunctiva.

These granulomas occur in any age group but most commonly in children, adolescents, and pregnant women due to the hormonal changes [3, 6]. They are characterized by a rapidly growing, pinkish‐red colored, smooth, sessile, or pedunculated lesion that is prone to ulceration and bleeding and occurs at the site of conjunctival injury. They can cause pain, discomfort, and irritation to the patient.

## 2. Case History and Examination

A 47‐year‐old female patient, without any underlying disease or history of current pregnancy, complained of irritation, discomfort, and redness in her left eye with a fleshy growth. Upon visiting the Ophthalmology Outpatient Department, she was diagnosed with Grade 2 inflamed pterygium and was advised to undergo pterygium excision surgery with conjunctival autologous graft.

The surgery proceeded smoothly without any intraoperative or postoperative complications. The conjunctival graft was sutured with Vicryl 8‐0 sutures and the suture was removed 10 days after the operation. Postsurgery medications included a tapering dose of steroid eye drops (1% prednisolone acetate) with an antibiotic eye drop (ciprofloxacin) and chloramphenicol eye ointment. The patient was followed up on the 10th day and 1 month after the operation. No complications or recurrence were noted during these follow‐up visits.

**Figure 1:**
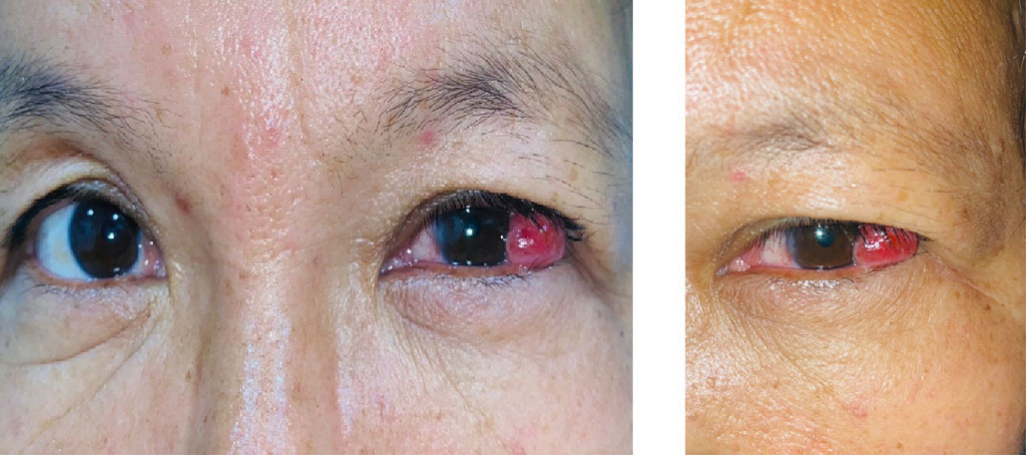
Conjunctival granuloma in the left eye of the patient: a pedunculated reddish lesion extending from the supero‐temporal conjunctiva and extending laterally was noted.

Then, 2 months after her initial surgery, the patient felt discomfort with irritation again and noticed an increasing lump in the same operated eye. Upon examination, her visual acuity was 6/6 in both her eyes and a pedunculated reddish lesion, extending from the supero‐temporal conjunctiva and extending laterally (Figure 1) was noticed in her left eye. There was no ulceration but minimal bleeding was noted. This lesion appeared at the site where the conjunctival graft was harvested during the previous surgery. A diagnosis of conjunctival pyogenic granuloma was made and she was planned for surgery to excise the granuloma but she refused the surgery, so medical treatment was opted first.

She was treated with 1% prednisolone acetate eye drops four times a day for a week and tapered to three times a day for a week, along with 0.5% timolol eye drops twice a day for 2 weeks and was advised to review after 2 weeks.

Despite the medical treatment for 2 weeks, the mass did not reduce in size and the symptoms of discomfort and irritation persisted so she was posted for surgical excision. The mass was excised and cauterization was done at the site of excision. Thorough cleaning of the surgical wound site was performed. The tissue was sent for pathological examination.

**Figure 2:**
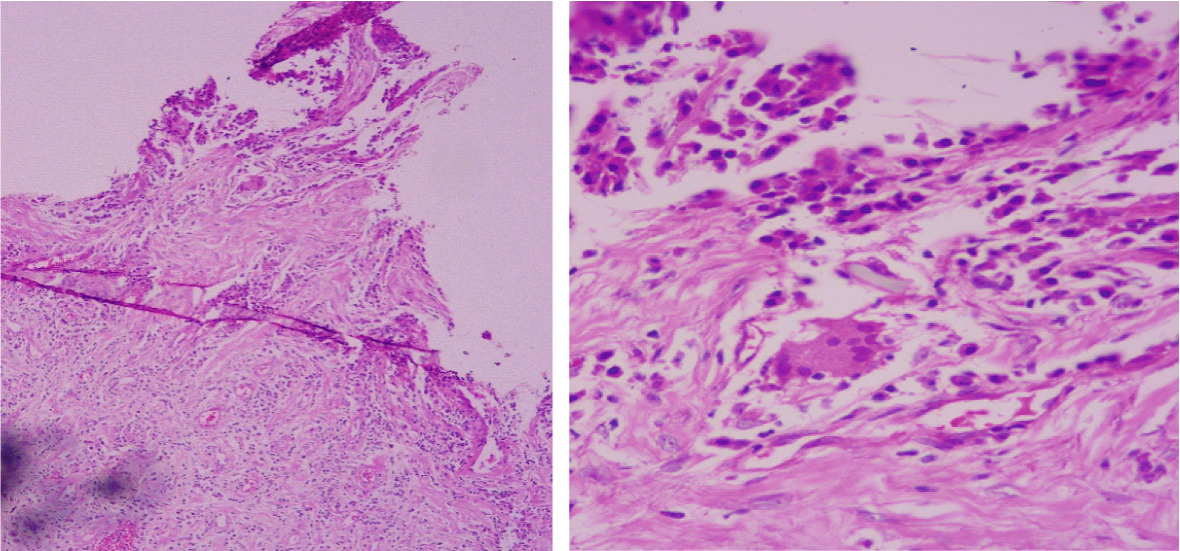
Histopathological aspect of the lesion (10x and 40x magnification): a conjunctival tissue lined by nondysplastic stratified squamous epithelium with core containing dense infiltrates of chronic inflammatory cells, few multinucleated giant cells, and a few refractile foreign body material.

The histopathological examination confirmed the diagnosis of conjunctival foreign body granuloma (Figure 2). The findings of the biopsy report showed a polypoid piece of conjunctival tissue lined by nondysplastic stratified squamous epithelium. The core contained dense infiltrates of chronic inflammatory cells with few multinucleated giant cells and a few refractile foreign body material. No malignancy was noted.

**Figure 3:**
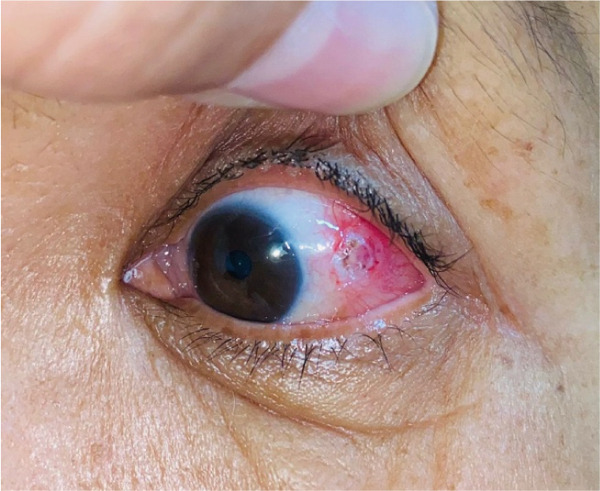
1 day after surgery with cauterized wound.

**Figure 4:**
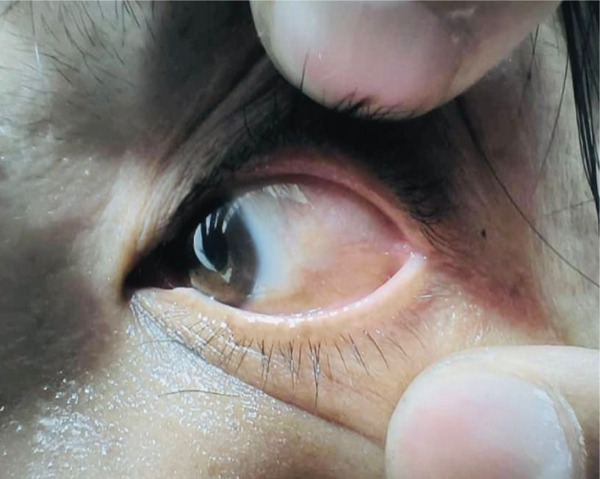
Postsurgery 1 month, wound healed, no recurrence, or abnormal growth noted.

She was reviewed immediately the next day for dressing and any abnormal bleedings (Figure 3) and followed up 1 month after the surgery (Figure 4). Apart from minimal scarring, no recurrence or any abnormal lesions were noted.

## 3. Discussion

Conjunctival pyogenic granulomas have no ethnic or gender preferences and can occur at any age, though they are most commonly seen in younger ages. They occur after eye trauma or eye‐related surgeries and during pregnancy due to hormonal changes. In some cases, the cause remains unknown.

In this particular case, the granuloma developed following pterygium surgery. While some granulomas do not have a clear cause and can arise without any insult to the conjunctiva, this patient′s condition was likely due to the previous surgical procedure causing improper wound healing or due to incomplete removal of the suture materials or other debris from the eye after surgery. A few cases of a pyogenic granuloma arising after pterygium surgery were reported in India as well [4].

Pyogenic granulomas post eye surgery are most commonly associated with the use of nonabsorbable sutures during the eye surgery; however, studies have shown that the granulomas can occur with absorbable sutures as well, though the inflammatory reactions with absorbable sutures are considered less severe than the nonabsorbable sutures [7]. In this case, an absorbable vicryl suture was used, which has caused the inflammatory reactions and the formation of the granuloma as seen in this patient.

Smaller granulomas may not have significant symptoms and are self‐limiting but larger lesions will cause pain, discomfort, irritation, interfere with the blinking leading to symptoms of dry eyes, and some may have an increased tendency to bleed and ulcerations of the granulomas causing infections. They can present as a pinkish‐red lesion, small, raised, or pedunculated arising from the site of the improper wound healing or as a reaction to a foreign body embedded in the conjunctiva after a trauma or an injury to the eye. They are nonmalignant lesions but can recur. Pyogenic granulomas are regarded as a misnomer because there is neither pus collection nor the formation of the granuloma, but they are formed due to an inflammatory response to a foreign body or a conjunctival injury [3].

On gross examination, they appear as pinkish‐red polypoid or pedunculated masses. Histopathologically, the lesions will have features of granulation tissue with inflammatory cells, and the presence of capillary proliferation will be noted [5, 8]. These lesions will spontaneously resolve at younger ages or if the granuloma is smaller in size. However, larger conjunctival granulomas often require medical or surgical interventions as they do not resolve spontaneously.

Since larger granulomas interfere with blinking, the most common complication that is seen is dry eyes, and eye infections are also possible due to the ulceration of these lesions.

Treatment options for nonresolving granulomas include topical medical therapy with steroids and timolol eye drops [3, 5, 8]. If medical treatment fails, surgical excision with cryotherapy and electrocautery is advised [5]. A surgical excision was necessary for this case due to lack of response to the medical treatment.

In small children or noncooperative patients, medical treatments can be opted as the first line of treatment. Studies have shown that conjunctival granulomas can resolve with the use of timolol eye drops which are selective beta‐blockers [3, 8, 9]. Timolol eye drops cause vasoconstriction of the blood vessels in the granulomas leading to their atrophy and the regression of the lesion. A study conducted by Nair AG et al. demonstrated that 91.6% of cases achieved complete resolution of the pyogenic granulomas with timolol eye drops [9] with a similar regression noted in a giant pyogenic granuloma post strabismus surgery [3]. These studies suggested that timolol eye drops could be considered as a potential treatment option for these kinds of lesions.

## 4. Conclusions

In summary, though conjunctival pyogenic granulomas arising from foreign bodies is not a new topic, this case highlights the importance and the need for meticulous surgical techniques and intraoperative care during pterygium excision.

While pterygium surgery is a minor ophthalmic procedure, insufficient or inappropriate intraoperative care, particularly the failure to completely remove residual debris or suture materials from the eyes, can result in complications such as the development of a foreign body granuloma similar to this case. Such practices can not only prolong the healing process but also increase the physical and emotional burden of the patient, requiring additional medical or surgical intervention.

## Ethics Statement

No ethical approval was required to report individual cases, but informed consent was taken from the patient.

## Consent

Written informed consent was obtained from the patient for the publication of this case report.

## Conflicts of Interest

The authors declare no conflicts of interest.

## Funding

No funding was received for this manuscript.

## Data Availability

Data sharing is not applicable to this article as no datasets were generated or analyzed during the current study.
